# Use of an AI Image Recognition–Based Smartphone App in Personalized Bowel Preparation Before Colonoscopy: Prospective Randomized Controlled Trial

**DOI:** 10.2196/77701

**Published:** 2026-07-14

**Authors:** Fei Han, Shilin Gao, Hanqiang Zhan, Nan Wang, Zhaoxiang Song, Jinlin Xie, Yuhan Hou, Yimeng Deng, Jianning Yao

**Affiliations:** 1 The First Affiliated Hospital of Zhengzhou University Zhengzhou China

**Keywords:** bowel preparation, colonoscopy, artificial intelligence, AI, smartphone

## Abstract

**Background:**

Sufficient bowel preparation is critical for increasing the quality of colonoscopy. However, current bowel preparation guidelines have limitations. We used a constructed and validated convolutional neural network model to assist patients in assessing the adequacy of bowel preparation and guide the use of laxatives based on individual variations.

**Objective:**

This research intended to explore the influence of AI-guided bowel preparation on patients’ use of laxatives and bowel cleansing quality.

**Methods:**

We conducted a prospective randomized controlled trial that included patients who had a colonoscopy at the First Affiliated Hospital of Zhengzhou University and used sodium phosphate solution for bowel cleansing. Included individuals were randomly categorized into 2 groups: the AI-guided group, which used an AI program to assess the adequacy of bowel cleansing and stopped the use of laxatives once the assessment was passed, and the control group, which evaluated bowel preparation adequacy based on standard practices and used the recommended doses of laxatives. The primary outcomes were the amount of laxatives used and the rate of adequate bowel cleansing based on the Boston Bowel Preparation Scale (BBPS). Other outcomes included overall BBPS scores, BBPS scores by colon region, adenoma detection rate (ADR), polyp detection rate, and advanced ADR, as well as adverse reactions and compliance.

**Results:**

This study included 513 patients (AI-guided group: n=255, 49.7%; control group: n=258, 50.3%). The volume of bowel-cleansing solution consumed by patients in the AI-guided group was 1245.5 (SD 377.2) mL, significantly lower than the 1586.2 (SD 50.4) mL in the control group (*P*<.001). Reduced use of laxatives was independently predicted by younger age, lower BMI, and higher defecation frequency. The rate of adequate bowel preparation in the AI group was not inferior to that in the control group (238/255, 93.3% vs 229/258, 88.8%, respectively; 1-sided *P*<.001 for noninferiority). No significant differences were observed in segmental or total BBPS scores, ADR, polyp detection rate, and advanced ADR between the 2 groups. Additionally, the medication adherence rate in the AI-guided group was significantly higher than in the control group (247/255, 96.9% vs 236/258, 91.5%, respectively; *P*=.009), whereas the incidence of adverse reactions was notably lower (5/255, 2% vs 15/258, 5.8%, respectively; *P*=.02).

**Conclusions:**

Using the AI-driven bowel preparation program to assist patients in personalized bowel preparation can reduce unnecessary use of laxatives while achieving results comparable to those of standard practices.

**Trial Registration:**

ClinicalTrials.gov NCT06610630; https://clinicaltrials.gov/study/NCT06610630

## Introduction

Colorectal cancer (CRC) is a malignant neoplasm that originates from the epithelial lining of the colon and rectum, with incidence and mortality rates rising each year [1]. Approximately 90% of CRCs develop from colorectal polyps, particularly adenomatous polyps [2,3]. If polyps are removed during the polyp stage via colonoscopy, this can prevent 70% to 90% of CRCs [4,5]. However, there is currently a high rate of missed polyps during colonoscopy. Research indicates that the miss rate for polyps after colonoscopy might reach 22% to 28%, with adenoma miss rates ranging from 12% to 26% [6]. These missed polyps and adenomas increase the risk of developing CRC [7,8]. Thus, it is evident that high-quality colonoscopy is essential for preventing CRC [9].

Quality control indicators for colonoscopy encompass adenoma detection rate (ADR), withdrawal duration, cecal intubation rate, and bowel preparation quality [10,11]. Among these, sufficient bowel preparation is fundamental for enhancing colonoscopy quality [12]. Although guidance through images and patient education can generally meet the requirements for bowel preparation, 20% to 25% of patients still have inadequate bowel preparation [13,14]. The underlying reason is that the adequacy of bowel preparation currently relies heavily on the patients’ subjective judgment, which can vary between observers. Therefore, a more objective and consistent assessment of bowel preparation, along with personalized guidance, is urgently needed in clinical practice.

AI has emerged as an auxiliary tool for this purpose. AI applications can use image recognition technology to assess bowel preparation adequacy in real time through a smartphone, allowing for objective assessment of bowel preparation and personalized guidance for cleansing. This approach aims to improve patients’ bowel preparation and optimize their cleansing experience. In this study, we hypothesized that not all patients need to consume the recommended doses of laxatives to achieve adequate bowel preparation. We used a developed AI platform to evaluate the adequacy of bowel cleansing in real time. Once the assessment was passed, the use of laxatives was stopped. This study sought to investigate the use of laxatives under AI direction and evaluate its impact on the quality of bowel preparation in patients.

## Methods

### Study Design

This was a prospective randomized controlled trial conducted from October 2024 to March 2025 at the First Affiliated Hospital of Zhengzhou University. This study included patients undergoing colonoscopy who were using sodium phosphate for bowel preparation. This study was registered in ClinicalTrials.gov with the registration number NCT06610630.

### Ethical Considerations

This study was approved the First Affiliated Hospital of Zhengzhou University’s ethics committee (2024-KY-1219-001) and aligned with the Declaration of Helsinki. All participants provided written informed consent prior to enrollment via a form that explicitly outlined study objectives, procedures, potential risks and benefits, and participants’ unrestricted right to withdraw at any time without penalty. All participant data were fully anonymized before analysis to protect confidentiality. Participants did not receive any financial compensation or other incentives for taking part in this study.

### Study Population

The research population consisted of individuals who had painless colonoscopy at the First Affiliated Hospital of Zhengzhou University from October 2024 to March 2025 using sodium phosphate for bowel preparation. Patients were recruited from the appointment registration office of the Digestive Endoscopy Center. During the study period, patients scheduled for painless afternoon colonoscopy and prescribed sodium phosphate solution for bowel preparation were systematically screened by trained research staff according to the predefined inclusion and exclusion criteria. Eligible patients must (1) use sodium phosphate for bowel cleansing, (2) perform bowel preparation in the morning and undergo colonoscopy in the afternoon, and (3) be able to use a smartphone autonomously or with help. The ability to use a smartphone was assessed during participant screening by trained staff. After standardized verbal instructions and a brief demonstration of the AI program, participants were evaluated on whether they, either independently or with assistance from an accompanying family member or caregiver, could complete the key steps required for using the program, including scanning the QR code, taking stool photographs, uploading images, and checking the feedback results. Patients who were unable to complete these steps and had no available assistant were considered unable to use the smartphone-based program. Individuals were excluded from the study if they refused to participate or met one or more of the following conditions: (1) contraindications to oral sodium phosphate solution, such as renal failure, congestive heart failure, bowel obstruction, congenital megacolon, or ascites; (2) allergy to components of sodium phosphate; and (3) history of colorectal surgery.

### AI Bowel Preparation Assessment Program

We used an AI platform developed based on a convolutional neural network (CNN) model accessible via QR code scanning with a mobile phone and provided by Xiamen Innovision Medical Technology in China. The CNN model was constructed for the intelligent evaluation of images of patients’ bowel preparation defecation. During the model training phase, a total of 4302 images were collected, and the size of the training set was determined through accuracy analysis. An incremental training strategy was adopted where 250 images were added to the training set each time, and the 10-fold cross-validation method was used to evaluate model performance under different sample sizes. The results showed that, with the increase in the number of training samples, model accuracy improved in stages: it increased rapidly in the initial stage, and when the sample size reached approximately 2500, accuracy increased to 97%. Thereafter, the slope of the curve approached 0, indicating that model performance gradually stabilized. Further verification demonstrated that, when the training set contained 3000 images or more, model accuracy could stably exceed 95%, and the total number of images used for final modeling exceeded 4000 [15]. To further evaluate its diagnostic performance, the model was tested on an independent dataset of 750 images that was not used for model training. The CNN model achieved a sensitivity of 93.02%, specificity of 92.56%, and overall accuracy of 92.80% for binary classification of bowel preparation images, supporting its reliability for real-time assessment of bowel cleansing adequacy.

Image annotation was completed by a nurse with 20 years of clinical experience who uniformly labeled the images to classify them into 2 categories: “qualified” (passing) or “unqualified” (not passing). During model initialization, parameters were randomly configured; in the training process, annotated images were input into the network to iteratively optimize the parameters. After training, the CNN model could realize automatic binary classification of newly uploaded bowel preparation images. To enhance clinical practicality, this study encapsulated the CNN model into a web service based on the MobileNetV3 architecture so as to meet the simultaneous use needs of a large number of patients and achieve real-time provision evaluation results. Compared with other CNN architectures, MobileNetV3 effectively addresses the efficiency bottleneck of traditional classification models through a lightweight network design (smaller scale and fewer parameters).

During use, after scanning the QR code, the system displays examples of how to take a photo of the stool and instructions for uploading the image to the system. After the image is uploaded, patients automatically receive an evaluation outcome of “pass” or “not pass.” Regarding those who have a “not pass” outcome, the system provides prompts to guide them in taking remedial measures, such as increasing physical activity, gently massaging the abdomen, and increasing fluid intake to enhance the efficacy of bowel cleansing.

### Bowel Preparation

Unblinded researchers used the SPSS (version 26.0; IBM Corp) software to generate the block randomization sequence with a block size set to 4. The allocation results were stored by independent statisticians (nonresearch staff). Independent third-party personnel prepared opaque sealed envelopes numbered by ID (containing group assignment slips) and delivered them to staff at the endoscopy registration office. The researchers had no prior knowledge of the group allocations. Staff at the appointment registration office randomly assigned participants to the AI-guided group or control group based on the allocation results, with a 1:1 allocation ratio between the 2 groups. All participants were given a bowel preparation instruction sheet when scheduling their colonoscopy, and trained physicians provided verbal explanations of the content, including recommendations for a low-fiber diet prior to the procedure, instructions for preparing the laxative solution, and methods for assessing the sufficiency of bowel preparation. All participants adhered to a low-fiber diet the day before bowel preparation and used sodium phosphate oral solution for cleansing. The standard sodium phosphate bowel preparation protocol is as follows: the first bowel preparation is scheduled for 8 AM, during which 45 mL of sodium phosphate solution are diluted in water to prepare approximately 800 mL of bowel-cleansing solution, which should be consumed within 30 minutes. The second dose is administered at 10 AM using the same method. After starting bowel preparation, patients in the AI group uploaded images of their stool using the app each time they had a bowel movement. Once the stool image was uploaded, the app displayed the assessment results as follows. On the basis of the uploaded image, a “pass” result was given if the assessment indicated that the preparation met the requirements for the procedure, and patients could proceed to the hospital for their appointment. An assessment of “not pass” was given when, based on the uploaded image, the assessment indicated that the preparation did not meet the requirements. Recommendations included increasing physical activity; gently massaging the abdomen in a clockwise direction; and, if necessary, increasing the use of laxatives. Patients were instructed to take another photo in their next bowel movement for reassessment until they received a “pass” result. Once a “pass” result was determined, patients were advised to stop taking the laxatives. In the control group, individuals assessed their bowel preparation based on the provided instructions without the assistance of the AI app.

### Data Collection

The primary outcomes were the actual volume of bowel-cleansing solution consumed and the proportion of patients achieving adequate bowel preparation. The volume of bowel-cleansing solution was recorded as the total amount of prepared sodium phosphate solution ingested before colonoscopy. Reduced laxative use was defined as consumption of at least 25% less than the standard 1600-mL bowel-cleansing solution. Endoscopists and nurses were completely unaware of the group assignments. Outcome assessors—specifically, 2 independent endoscopists—had no access to group information, and patients were only exposed to the interventions designated for their respective groups. Two endoscopists evaluated the patients’ bowel preparation status using the Boston Bowel Preparation Scale (BBPS) [12,16]. The ultimate decision was made by a senior endoscopist if they could not come to an agreement. The definition of sufficient bowel preparation was a total BBPS score of 6 or higher, with each segment score being 2 or higher [17]. Secondary outcomes included total BBPS score, segmental BBPS scores, polyp detection rate (PDR), ADR, advanced ADR (aADR), medication adherence, patient-reported acceptance of the AI program, and adverse events. PDR was defined as the proportion of patients with at least one detected polyp, and ADR was defined as the proportion of patients with at least one detected adenoma. Advanced adenomas were defined as adenomas larger than 10 mm, those with high-grade dysplasia, or those with villous features [18]. Medication adherence in the AI-guided group was defined as following the AI program instructions, including discontinuing medication after receiving a “pass” result or implementing remedial measures after receiving a “not pass” result. Medication adherence in the control group was defined as completing the full prescribed dose of bowel-cleansing solution, namely, 1600 mL. Patient-reported acceptance of the AI program was assessed in the AI-guided group based on whether patients could successfully use the program without additional instructions and whether they were willing to use the AI program again for future bowel preparation. Adverse events were recorded through patient self-report and structured inquiry before colonoscopy. These included symptoms occurring during bowel preparation, such as nausea, vomiting, abdominal pain, bloating, dizziness, and palpitations. The incidence of adverse events was defined as the proportion of patients reporting at least one of these symptoms. Regarding clinical characteristics, daily defecation frequency was categorized into 3 levels: low frequency (fewer than 3 times per week), medium frequency (2 times per day to 3 times per week), and high frequency (more than 2 times per day).

### Statistical Analysis

The sample size was calculated based on data from previous studies and pilot test results. We set the adequate bowel preparation rate for the control group at 85% assuming an adequate bowel preparation rate of 90% for the AI group. The noninferiority margin was set at −5 percentage points. Using PASS (version 11; NCSS, LLC) for calculations, we established that a sample size of 230 per group would achieve 90% statistical power and a significance level of .025 to detect noninferiority between the groups. Considering a 10% attrition rate, at least 512 patients were required. Data analysis was conducted using SPSS (version 26.0) and R (version 4.5.0; R Foundation for Statistical Computing). Categorical data were reported as counts and percentages, and the chi-square test, continuity-corrected chi-square test, or Fisher exact test were used to compare the 2 groups. For continuous variables, normal distribution was represented as means and SDs, whereas skewed distribution was reported as medians with IQRs. Group comparisons were made using *t* tests, Welch *t* tests, or nonparametric tests (Mann-Whitney *U* tests). A 2-tailed *P* value of less than .05 was considered statistically significant for conventional between-group comparisons. We used a one-sided test to evaluate the noninferiority of AI-guided bowel preparation vs the control group. Noninferiority was concluded if the lower bound of the 95% CI for the difference in adequate bowel preparation rate did not cross the –5–percentage point margin. To find predictors of reduced laxative use in the AI-guided group, logistic regression analysis was used.

## Results

### Patient Characteristics

**Figure 1:**
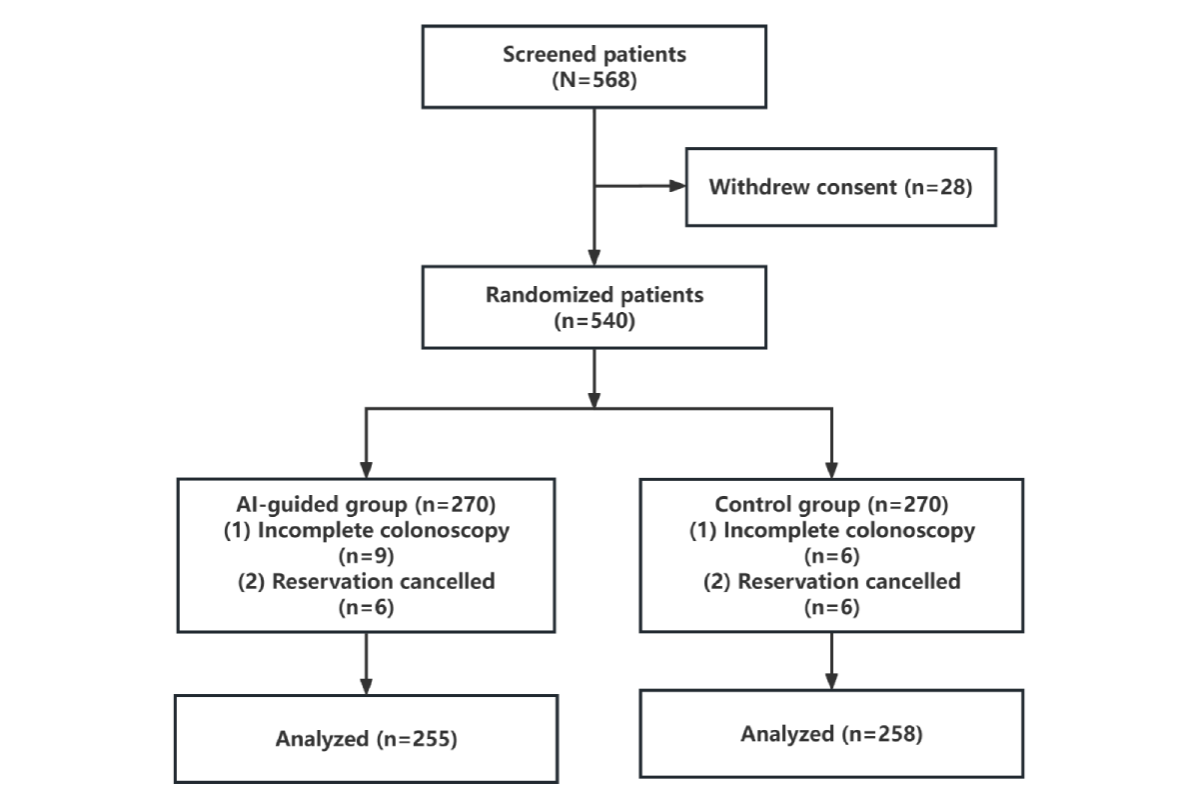
Flow diagram for patient inclusion.

A total of 540 patients were randomly assigned to both groups, of whom 27 (5%) were excluded due to incomplete cecal intubation for reasons such as rectal cancer. Ultimately, the study comprised a total of 513 patients (n=255, 49.7% in the AI-guided group and n=258, 50.3% in the control group; Figure 1). There were no significant variations among the 2 groups’ baseline features, including age, sex, BMI, indications for colonoscopy (screening, surveillance, and diagnosis), daily defecation frequency, and relevant medical history (history of abdominal surgery, coronary heart disease, diabetes, and hypertension; Table 1).

**Table 1:** Patient baseline characteristics.

			AI-guided group (n=255)		Control group (n=258)		*P* value	
Age (y), mean (SD)			47.77 (13.20)		49.53 (11.81)		.11	
Male, n (%)			125 (49)		142 (55)		.17	
BMI (kg/m^2^), mean (SD)			23.84 (2.75)		24.33 (3.08)		.06	
**Colonoscopy indication, n (%)**								.84
	Screening	81 (31.8)		77 (29.8)				
	Surveillance	66 (25.9)		72 (27.9)				
	Diagnostic	108 (42.4)		109 (42.2)				
**Defecation frequency, n (%)**								.39
	Low	52 (20.4)		41 (15.9)				
	Medium	181 (71)		191 (74)				
	High	22 (8.6)		26 (10.1)				
Previous surgery (abdominal or pelvic), n (%)			50 (19.6)		61 (23.6)		.27	
**Medical history, n (%)**								
	Diabetes	36 (14.1)		40 (15.5)		.66		
	Hypertension	51 (20)		59 (22.9)		.43		
	Coronary disease	17 (6.7)		19 (7.4)		.76		

### Volume of Laxatives Consumed

In the control group, all participants (258/258, 100%) consumed the recommended dosage of sodium phosphate solution for bowel preparation. In the AI-guided group, 48.6% (124/255) of the patients reduced their use of laxatives. The average volume of laxatives consumed in the AI-guided group was 1245.5 (SD 377.2) mL, which was significantly lower than the 1586.2 (SD 50.4) mL consumed in the control group (*P*<.001). The multivariate analysis revealed that reduced use of laxatives was independently predicted by younger age, lower BMI, and higher defecation frequency (Table 2).

**Table 2:** Predictive factors for reducing the use of laxatives.

			Univariate analysis				Multivariate analysis		
			OR^a^ (95% CI)		*P* value		OR (95% CI)		*P* value
Age			0.957 (0.937-0.976)		*<.001* ^b^		0.964 (0.942-0.986)		*.001*
**Sex**									
	Male	1		—^c^		—		—	
	Female	1.191 (0.729-1.948)		.49		—		—	
BMI			0.719 (0.642-0.805)		*<.001*		0.739 (0.656-0.832)		*<.001*
**Previous surgery (abdominal or pelvic)**									
	Yes	1		—		—		—	
	No	1.713 (0.909-3.227)		.10		—		—	
**Defecation frequency**									
	Low	1		—		1		—	
	Medium	3.241 (1.622-6.476)		*.001*		2.595 (1.209-5.572)		*.01*	
	High	10.200 (3.140-33.136)		*<.001*		10.200 (3.365-43.729)		*<.001*	
**Diabetes**									
	Yes	1		—		—		—	
	No	1.586 (0.772-3.260)		.21		—		—	
**Hypertension**									
	Yes	1		—		—		—	
	No	1.194 (0.645-2.210)		.57		—		—	
**Coronary disease**									
	Yes	1		—		—		—	
	No	0.493 (0.177-1.377)		.18		—		—	

^a^OR: odds ratio.

^b^Italics indicate statistical significance.

^c^Not applicable.

### Bowel Preparation Quality and Colonoscopy Results

No significant difference was observed in the proportion of patients achieving sufficient bowel preparation between the AI-guided group and the control group (238/255, 93.3% vs 229/258, 88.8%, respectively; *P*=.07). The noninferiority analysis demonstrated that the AI-guided group was noninferior to the control group as the CI did not exceed the noninferiority margin of –5 percentage points and the one-sided *P* value was statistically significant (*P*<.001; 95% CI −0.0038 to 0.0971). Additionally, the average BBPS scores for each colon region, ADR, PDR, and aADR did not differ significantly between the 2 groups (Table 3).

**Table 3:** Quality of bowel preparation and colonoscopy outcomes in both groups.

			AI-guided group (n=255)		Control group (n=258)		*P* value	
**Bowel preparation quality, n (%)**								.07
	Adequate	238 (93.3)		229 (88.8)				
	Inadequate	17 (6.7)		29 (11.2)				
**BBPS^a^** **score, mean (SD)**								
	Total	7.44 (1.20)		7.26 (1.22)		.09		
	Right colon	2.23 (0.56)		2.14 (0.57)		.07		
	Transverse colon	2.68 (0.47)		2.68 (0.47)		>.99		
	Left colon	2.53 (0.50)		2.44 (0.55)		.06		
Adenoma detection rate, n (%)			89 (34.9)		88 (34.1)		.85	
Polyp detection rate, n (%)			112 (43.9)		116 (45.0)		.81	
Advanced adenoma detection rate, n (%)			16 (6.3)		8 (3.1)		.09	

^a^BBPS: Boston Bowel Preparation Scale (each colon segment of the BBPS ranges from 0 to 3, and the total BBPS score ranges from 0 to 9, with higher scores indicating better bowel preparation quality).

### Patient-Reported Acceptance, Medication Adherence, and Incidence of Adverse Events

A total of 94.9% (242/255) of patients were able to successfully use the AI bowel preparation program without additional instructions. In total, 96.5% (246/255) of patients expressed a willingness to use the AI bowel preparation program for their next bowel cleansing. In addition, the medication adherence rate in the AI-guided group was significantly higher than that in the control group (247/255, 96.9% vs 236/258, 91.5%, respectively; *P*=.009), whereas the incidence of adverse reactions was notably lower (5/255, 2% vs 15/258, 5.8%, respectively; *P*=.02). Specifically, the incidence of reported adverse reactions in the AI-guided group vs the control group was as follows: 1.6% (4/255) vs 4.7% (12/258) for nausea (*P*=.045), 1.2% (3/255) vs 3.9% (10/258) for vomiting (*P*=.052), and 1.2% (3/255) vs 4.7% (12/258) for bloating (*P*=.02).

## Discussion

In this research, we used a developed and validated AI bowel preparation program to assess stool status in real time and guide patients in personalized bowel cleansing. In the AI-guided group, 48.6% (124/255) of the participants reduced the use of laxatives. The proportions of adequate bowel cleansing, BBPS scores, and ADR did not vary significantly between the AI-guided group and the control group. This suggested that AI-guided bowel cleansing can provide patients with a more personalized bowel preparation plan, reducing unnecessary use of laxatives.

In previous studies, to help patients prepare their bowels better, a number of therapies have been investigated. For example, visual aids [19] and educational videos [20] have been used to guide bowel cleansing in a more understandable manner. Additionally, patient education has been reinforced through phone calls [21,22] or smartphone apps [23-25] to enhance bowel preparation quality. However, these studies have primarily focused on enhancing patient education and do not provide real-time assistance for patients to assess the sufficiency of their bowel preparation. In recent years, research has suggested that AI-driven smartphone apps can evaluate bowel preparation adequacy in real time, thereby improving patients’ bowel preparation [26,27]. However, these studies have only focused on how AI programs can provide recommendations for additional laxative use for patients with inadequate bowel preparation. Different individuals may respond differently to laxatives. For instance, patients with constipation or older adults may require more laxatives, whereas those with normal bowel habits or irritable bowel syndrome may achieve adequate preparation with less. Currently, limited research has explored the feasibility of an AI bowel preparation program providing tailored recommendations for increasing or decreasing laxative use based on individual responses.

We proposed a new approach for bowel preparation based on an AI bowel preparation program. After a successful AI assessment, patients could stop taking laxatives, whereas those who did not pass would take additional remedial measures to achieve adequate preparation. The final results showed that participants in the AI-guided group could perform bowel preparation in a way comparable to conventional practices after consuming an average of approximately 1.25 L of bowel-cleansing solution. This significantly reduced the volume of cleansing solution used compared to the 3-L or 4-L polyethylene glycol solution or the 1.6-L sodium phosphate solution recommended by guidelines and other studies [28-30]. This suggests that the AI bowel preparation program has significant potential for guiding individualized laxative use. In the logistic regression analysis, younger age, lower BMI, and higher defecation frequency were identified as independent predictive factors for reduced laxative use. Future research could establish a predictive model for bowel-cleansing agent dosage by increasing the sample size and considering other relevant factors, providing patients with reasonable laxative use plans in situations in which using AI programs is not feasible.

In terms of bowel preparation quality across the 2 groups, the AI-guided group exhibited superior rates of sufficient bowel preparation and BBPS scores compared to the control group. However, these differences lacked statistical significance. Despite thorough explanations and education, some patients may not have used the AI bowel preparation program correctly. Data indicated that 1.2% (3/255) of the patients took a photo of an empty toilet bowl, which could have led to inaccuracies in the AI assessment. Additionally, some patients who did not pass the assessment after taking the laxatives failed to follow the recommended remedial measures for bowel preparation. Ultimately, only 4.7% (12/255) of the patients undertook additional remedial actions and postponed their colonoscopy, whereas other patients who did not pass the assessment proceeded with their scheduled colonoscopy, which may have led to an underestimation of the effectiveness of the AI-guided program. The colonoscopy results, such as ADR, PDR, and aADR, did not exhibit substantial variations between the 2 groups. Previous studies have suggested that enhanced educational methods such as telephone or WeChat education could achieve higher ADR [21,23]. A possible reason for this is that bowel preparation quality was comparable in both groups, and the ADR among the endoscopists at our center was relatively high (89/255, 34.9% in the AI-guided group and 88/258, 34.1% in the control group), which may have led to an inability to observe differences in ADR and other colonoscopy outcomes.

In the AI-guided group, patients had a generally positive attitude toward the use of the AI program. Most were able to successfully use the AI program for bowel preparation after a simple explanation and introduction. Nevertheless, some older individuals were excluded from the study because they were unable to use smartphones or unable to use them with assistance from others. To address this issue, first, the future program interface should be designed to be more concise, with visual operation instructions to ensure that it is easy to understand and use. Second, for older patients who still cannot master smartphone operations, their immediate family members (or accompanying personnel) could be asked to assist with image uploads and feedback checks. Additionally, some patients experienced issues with the display of evaluation results due to network problems, but they successfully used the program after troubleshooting their network and devices. To address network obstacles, an “offline evaluation–subsequent synchronization” dual-mode approach should be developed in the future. The app should support locally storing encrypted images and perform offline analysis using lightweight CNNs, with automatic data synchronization enabled once network connectivity is restored. Regarding adverse reactions, AI-guided laxative dose reduction lowered the incidence of nausea, vomiting, and abdominal distension, with no severe adverse events reported. This benefit arose from the AI model’s ability to precisely identify optimal bowel cleanliness thresholds through real-time image analysis, thereby avoiding overmedication in nonconstipated patients under fixed-dose regimens. Mechanistically, this reduced intestinal distension and vagal nerve stimulation. Furthermore, patients in the AI-guided group had a higher medication adherence, indicating that the AI program has high usability in clinical practice and significant potential for broader application.

This research has limitations. First, this study exclusively included patients using sodium phosphate for bowel preparation, a decision driven by our research design. However, this approach may restrict the broader applicability of our findings to other laxatives (eg, polyethylene glycol). Future studies should expand the sample size and incorporate multiple laxative regimens to enhance the clinical generalizability of AI-driven bowel cleanliness assessment tools. Second, the operation of the AI bowel preparation program relies on the support of smartphones and wireless networks, which means that some individuals who cannot use smartphones, particularly certain older patients, were excluded from the study, potentially leading to selection bias.

In conclusion, this study presents the use of an AI bowel preparation program to guide patients in personalized bowel preparation, which can reduce unnecessary use of laxatives while achieving results comparable to those obtained with standard practices. Promoting this program in clinical practice could help improve the efficiency and comfort of bowel preparation for patients.

## Appendix

Multimedia Appendix 1 CONSORT-EHEALTH checklist (V 1.6.1).

## Abbreviations

aADR: advanced adenoma detection rate
ADR: adenoma detection rate
BBPS: Boston Bowel Preparation Scale
CNN: convolutional neural network
CRC: colorectal cancer
PDR: polyp detection rate

## Data Availability

The data that support the findings of this study are available from the corresponding author on reasonable request.
